# Microfluidic fabrication of lipid-coated PLGA nanoparticles co-delivering CuS and curcumin for combined chemo-photothermal therapy

**DOI:** 10.3389/fbioe.2026.1805754

**Published:** 2026-10-28

**Authors:** Alhasan Mohammed, Yu Xia, Hemin Roghzai, Bo Zhang, Xiubo Zhao

**Affiliations:** 1 School of Pharmacy, Changzhou University, Changzhou, China; 2 College of Science, University of Sulaimani, Sulaymaniyah, Iraq

**Keywords:** cancer therapy, curcumin, drug delivery, lipid NPs, microfluidics, PLGA NPs

## Abstract

Cervical cancer remains a major global health challenge, with chemotherapy serving as a primary treatment modality. However, its clinical efficacy is often compromised by systemic toxicity, poor drug solubility, and non-specific targeting. To address these limitations, we report the fabrication of lipid-coated poly(lactic-co-glycolic acid) (PLGA) nanoparticles (NPs) co-delivering curcumin (Cur) and copper sulfide (CuS) for combined chemo-photothermal therapy using a microfluidic swirl mixer. The microfluidic platform enables controlled, reproducible preparation of the NPs with low polydispersity (PDI) and high co-encapsulation efficiency of both compounds. The primary innovation of this work lies in integrating a PLGA -lipid hybrid architecture with simultaneous encapsulation of a model anticancer drug Cur and an NIR-responsive photothermal agent, CuS into a single formulation. Optimization of the flow rate ratio (FRR) and total flow rate (TFR) enabled precise tuning of particle size and physicochemical properties. CuS/Cur@PLGA@Lipid NPs the sustained drug release from the PLGA core with the improved colloidal stability and cellular uptake provided by the lipid shell. In vitro evaluations demonstrated pH-sensitive release, with accelerated Cur liberation under acidic microenvironments relative to physiological conditions. Furthermore, the NPs exhibited pronounced cytotoxicity against cervical cancer cells (HeLa), which was significantly enhanced under NIR laser irradiation via CuS-driven photothermal ablation. Quantitative fluorescence and flow-cytometry analyses confirmed superior cellular uptake and intracellular retention compared to free Cur, while multicellular tumor spheroid models demonstrated enhanced three-dimensional antitumor efficacy. Overall, this study provides an in vitro proof-of-concept for the high-throughput potential for the production of lipid-coated PLGA NPs with dual chemo-photothermal therapeutic potential.

## 1 Introduction

Cervical cancer is the third leading cause of death among gynecological malignancies, with approximately 13,000 new cases of invasive cervical cancer diagnosed annually and a fatality rate near 30% (Sims et al., 2016). Although primarily caused by persistent high-risk Human Papillomavirus (HPV) infection (Athanasiou et al., 2022), it remains a severe global public health challenge, ranking as the fourth most common cancer in women worldwide—particularly affecting low- and middle-income regions.

Chemotherapy is a common treatment for cervical cancer. However, conventional chemotherapeutic agents suffer from poor solubility, instability, and rapid systemic clearance, leading to inefficient drug accumulation at the tumor site and systemic toxicity with short- and/or long-term side effects (Chen X. et al., 2024). Nano drug delivery systems (NDDSs) can passively accumulate in tumor tissues via the enhanced permeability and retention (EPR) effect, offering a strategy to improve tumor-specific delivery (Ejigah et al., 2022; Sun et al., 2023). Onivyde®, Doxil®, Myocet®, Caelyx® are perhaps the best-known examples (Arshad et al., 2023; Gonda et al., 2019), employing different NPs (e.g., liposomes and polymeric NPs) carrying a range of chemotherapeutic agents for targeted therapies, or even gene therapies.

Curcumin (Cur), a hydrophobic polyphenol extracted from the rhizomes of turmeric (Curcuma longa L.) (Hao et al., 2023; Kongpol et al., 2022), has gained significant attention for its multiple pharmacological activities, including antioxidant, anti-inflammatory, antibacterial, antiviral, and anti-tumor properties (Ashrafizadeh et al., 2020). Cur exhibits anti-cancer activity in cervical cancer by suppressing tumor cell growth and metastasis, triggering apoptosis, and strengthening the immune response (Hao et al., 2023; Hegde et al., 2023). However, the clinical application of Cur is limited by its poor bioavailability and rapid metabolism. NDDSs with controlled particle size and surface modifications can efficiently deliver drugs to tumor tissues (Amina et al., 2024). Poly (lactic-co-glycolic) acid (PLGA), a biodegradable copolymer, is widely used in DDSs due to its controllable degradation rate and plasticity (Tsung et al., 2023). It enables the preparation of various drug carriers, such as microspheres, NPs, and scaffolds, facilitating sustained release and targeted delivery of drugs. Lipid NPs are employed for mRNA delivery in viral vaccines, cancer immunotherapies, and the treatment of genetic diseases highlighting their versatility and potential. Outer lipid coatings (composed of phospholipids and cholesterol) can significantly improve colloidal stability, enhance cell membrane affinity, promote cellular uptake, and facilitate endosomal escape (M et al., 2018). These findings are consistent with recent developments in stimuli-responsive liposomal and polymeric nanocarrier systems, which further support the potential of engineered nanocarriers for controlled therapeutic delivery (Basak and Das, 2026; Basak et al., 2024; Basak and Das, 2025a; Basak and Das, 2025b).

To enable a combined chemo-photothermal therapeutic approach, copper sulfide (CuS) NPs were selected as the photothermal agent. CuS is a highly promising material for cancer therapy due to its strong near-infrared (NIR) absorbance, effective NIR-responsive photothermal heating, and favorable biocompatibility (Atmaca et al., 2022). Previous studies have demonstrated that at therapeutic concentrations, CuS NPs can be gradually biodegraded in the acidic tumor microenvironment into copper ions, which can be cleared by the body’s metabolic pathways. This biodegradability minimizes concerns about long-term accumulation and toxicity, making CuS a compelling choice for combination cancer therapy (Chen G. et al., 2024).

In this study, we developed a multifunctional, lipid-coated PLGA nanoplatform co-encapsulating Cur (as a hydrophobic model chemotherapeutic) and CuS NPs (as a photothermal agent) using a microfluidic swirl mixing technique. By systematically tuning the total flow rate (TFR) and flow rate ratio (FRR), this platform achieves precise control over particle size and low polydispersity index (PDI) in a rapid manner, with high-throughput and scalability potential (Roschenko et al., 2023; Xia et al., 2023). The streamlined preparation has the potential for large-scale production based on the throughput demonstrated in our previous work (up to 320 mL/min demonstrated in our previous work) (Xu et al., 2022; Tomeh et al., 2022a). The primary contribution of this work lies in integrating a microfluidic fabrication workflow with lipid-associated PLGA NPs, dual-cargo encapsulation (Cur and CuS), NIR responsiveness, and microfluidic process control into a single multifunctional strategy. The resulting optimized Cur@PLGA@Lipid NPs feature narrow size distribution, high encapsulation efficiency, pH-responsive drug release, robust photothermal performance, enhanced cellular uptake, and superior cytotoxicity across both 2D monolayer and 3D multicellular tumor spheroid models of HeLa cervical cancer. Collectively, these features supported the development of a versatile nanoplatform with potential applications in sustained release and potential combination cancer therapy.

## 2 Experimental section

### 2.1 Materials

PLGA (50:50, MW = 15,000) was obtained from the Jinan Daigang Bioengineering Co., Ltd (Shandong, China). Curcumin (Cur, MW 368.38 Da), absolute ethanol (EtOH) and acetonitrile (ACN) were purchased from Sinopharm Chemical Reagent Co., Ltd., Shanghai, China. Cholesterol (Chol) and Hydrogenated soy phosphatidylcholine (HSPC) were purchased from AVT (Shanghai) Pharmaceutical Technology Co., Ltd., Shanghai, China. Dulbecco’s modified Eagle’s medium (DMEM), fetal bovine serum (FBS) and penicillin/streptomycin were obtained from Gibco (Thermo Fisher Scientific), Grand Island, NY, United States. 3-(4,5-dimethylthiazol-2-yl)-2,5-diphenylte- trazolium bromide (MTT), 4′,6-diamidine-2′-phenylindole dihydro-chloride (DAPI), 2′-(4-Ethoxyphenyl)-5-(4-methyl-1- piperazinyl)-2,5′-bi-1H-benzimidazole, trihydrochloride (Hoechst 33342) and propidium iodide (PI) were purchased from Beijing Solarbio Bio-Tech Co. Ltd., Beijing, China. Phosphate buffer (PBS) and all other reagents used in this research were of analytical grade.

### 2.2 Preparation of copper sulfide nanoparticles (CuS NPs)

Copper chloride dihydrate (8.525 mg) and trisodium citrate (20 mg) were fully dissolved in a 100 mL single-necked flask with 45 mL of deionized (DI) water and stirred at 100 rpm. Sodium sulfide (10 mg) dissolved in 5 mL DI water was quickly added into the above solution with stirring speed at 800 rpm. The solution changed from colorless to light yellow. After 5 min, the solution was heated to 90 °C and maintained at high-speed stirring for 15 min. The solution will change from light yellow to light green and then to dark green, indicating the reaction is complete. The sample was cooled to room temperature, filtered through a 0.22 μm filter, and stored in a clean glass bottle at 4 °C for future use.

### 2.3 Preparation of PLGA NPs using microfluidics

The microfluidic device (Figure 1a) was developed by our own research group. A more detailed schematic diagram of the microfluidic device can be found in our previous report (Xia et al., 2023). PLGA was fully dissolved in acetonitrile with the help of ultrasonication and filtered with a 0.22 μm filter. A series of solutions (2.5, 5, 10, and 20 mg/mL) of PLGA were used as the organic phase and the DI water was used as the aqueous phase following previously optimized microfluidic nanoprecipitation protocols and introduced into one inlet of the microfluidic device using a Fusion 4000 syringe pump (ChemYX, Stafford, TX, United States) connected to a microfluidic swirl mixer chip. The aqueous phase (DI water) was introduced into the other inlet of the microfluidic device promoting rapid mixing and nanoprecipitation. The flow rate ratios (FRRs) were adjusted to 1:1, 1:2, 1:3, 1:5, 1:7 and 1:9 (organic: aqueous), respectively. The total flow rates (TFRs) were set at 5, 15, 30, 45, and 60 mL/min. The particle size, polydispersity index (PDI) and ζ-potential of the PLGA NPs were measured using a Malvern Ζetasizer (Nano-ZS, Malvern, United Kingdom) to optimize the experimental conditions.

**FIGURE 1:**
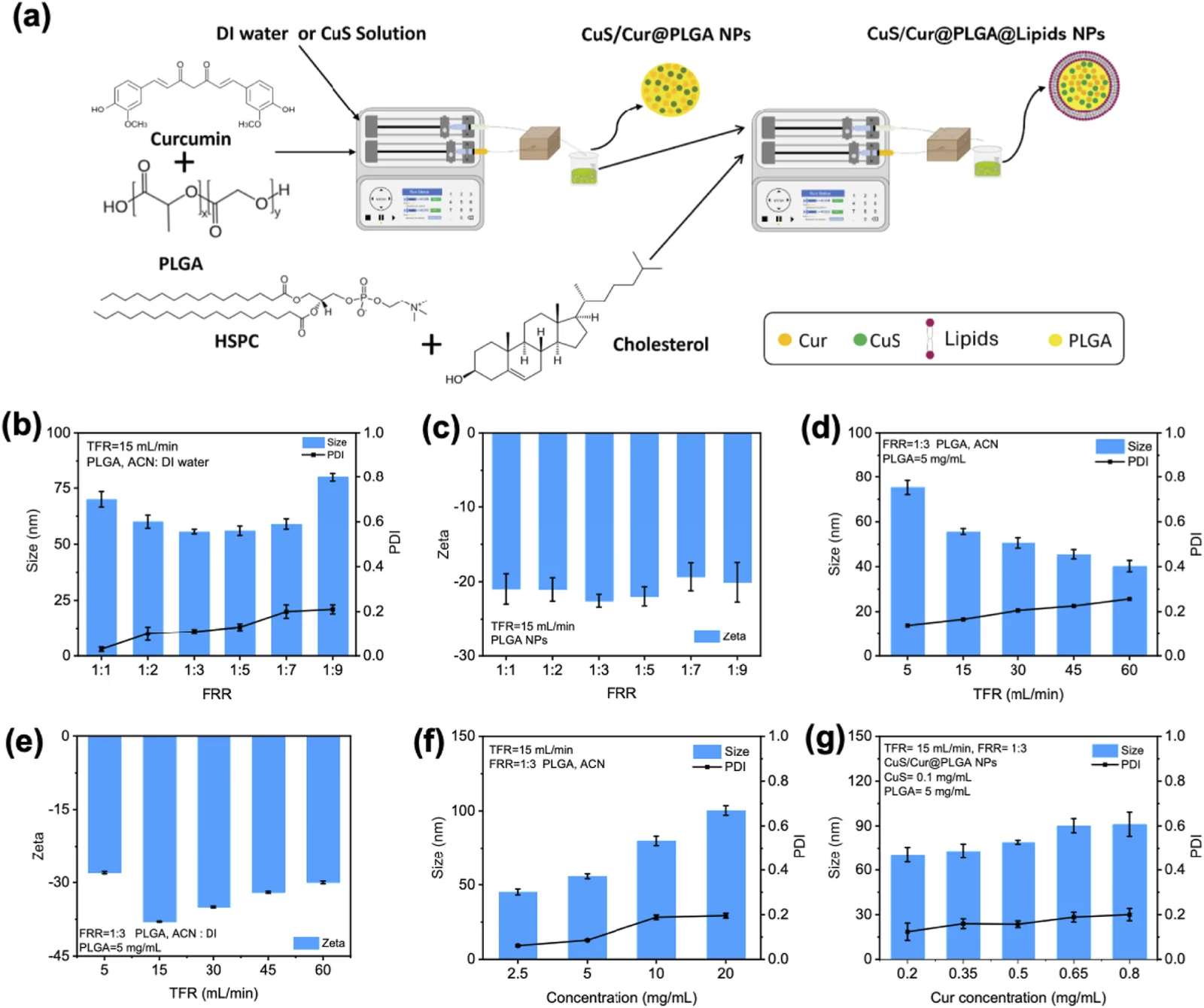
Microfluidic fabrication and optimization of PLGA NPs. **(a)** Schematic workflow of NP preparation. Effects of flow rate ratio on particle size/PDI **(b)** and ζ potential. **(c)** Effects of total flow rate on particle size/PDI **(d)** and ζ potential. **(e)** Effects of PLGA concentration **(f)** and curcumin concentration **(g)** on particle size and PDI. Data are presented as mean ± SD (n = 3).

### 2.4 Preparation of lipid NPs using microfluidics

HSPC and Chol were mixed at concentration ratios of 1:1, 1:2, 1:3, 1:4, 1:5, 1:7, and 1:9 dissolved in EtOH followed by ultrasonication. Lipid solutions served as an organic phase, and PBS as the aqueous phase (Hong et al., 2024; Zeng et al., 2024). The FRRs (organic: aqueous) were set to 1:1, 1:2, 1:3, 1:4, 1:7, and 1:9, respectively. The TFRs of the two phases were set at 5, 15, 30, and 40 mL/min. The two phases were pumped into the inlets of the microfluidic device as outlined previously for the preparation of lipid NPs. The particle size, PDI, and ζ potential of the lipid NPs was measured using a Malvern Ζetasizer (Nano-ZS) to optimize the experimental conditions.

### 2.5 Preparation of Cur@PLGA NPs using microfluidics

Curcumin-loaded PLGA NPs were synthesized using the microfluidic nanoprecipitation method with the swirl mixer. PLGA (5 mg/mL) and curcumin (0.5 mg/mL) were dissolved in ACN and filtered through a 0.22 μm membrane to form the organic phase. DI water or a copper sulfide solution was used as the aqueous phase. The organic and aqueous phases were pumped into the microfluidic mixer at a FRR of 1:3 (organic: aqueous), with a TFR of 15 mL/min to form Cur@PLGA NPs. Blank PLGA NPs were also prepared without curcumin or CuS. After NP formation, residual ACN was removed by evaporation.

### 2.6 Preparation of CuS/Cur@PLGA@Lipid NP using microfluidics

The CuS/Cur@PLGA NPs were synthesized by modifying the protocol described in Section 2.5. Briefly, the aqueous phase was replaced with a 0.1 mg/mL CuS solution during PLGA nanoprecipitation to incorporate CuS into the PLGA-based NP formulation. Lipid coating was performed through microfluidic mixing again after formation of the CuS/Cur@PLGA NPs. A phospholipid/cholesterol solution (HSPC:Chol = 2:1 M ratio in ethanol) was selected based on prior optimization studies (Rahni and Kazakov, 2017), while the purified CuS/Cur@PLGA NPs were dispersed in deionized water to serve as the aqueous phase (Shi et al., 2022). These two phases were introduced into the microfluidic device at a 1:4 FRR (organic: aqueous) with a TFR of 15 mL/min. The resulting dispersion was centrifuged at 10,000 rpm for 15 min to pellet the NPs. The supernatant containing unbound lipids was carefully decanted. The lipid coated NPs were washed twice with deionized water before resuspension. Final formulations were stored at 4 °C until use.

### 2.7 Characterization of CuS/Cur@PLGA@Lipid NPs

The particle sizes, PDI, and ζ potentials of blank PLGA NPs, blank lipid NPs, Cur@PLGA NPs, Cur@PLGA@Lipid NPs, and CuS/Cur@PLGA@Lipid NPs were determined using dynamic light scattering (DLS). Measurements were taken at 25 °C with a Ζetasizer (Nano-ZS, Malvern, United Kingdom). Each sample was measured three times, and the results were reported as mean ± standard deviation (SD). The morphologies of the NPs were observed through Nanowizard4 AFM using SCANASYST-AIR tips.

### 2.8 Drug encapsulation efficiency (EE) and loading capacity (LC)

The loading capacity (LC) and encapsulation efficiency (EE) of Cur were evaluated using a UV-vis spectrophotometer (TU-1901PC, Shanghai, China) at 425 nm wavelength. This wavelength was selected as corresponds to the absorption maximum of curcumin and is commonly used for NP formulations. While it is adequate for measuring concentration in this study, we acknowledge HPLC is a better option in future studies. Briefly, 2 mL of the drug-loaded PLGA NP solution was centrifuged at 15,000 rpm for 45 min and the supernatant was discarded. Then, 1 mL of acetonitrile was added to the precipitate and ultrasonicated. The solution was diluted to measure the absorbance value using the UV-Vis spectrophotometer. The mass m1 of the drug was calculated according to the standard curve. The total amount of Cur in the tube is m2, and the encapsulation efficiency of the drug-loaded NPs was calculated by Equation 1. The precipitate (from 2 mL solution) after centrifugation was dried overnight at 25 °C, and the mass of precipitate m3 was weighed. The drug loading was calculated according to Equation 2.

$$\text{EE}(\%)=\left(m_{1}/\;m_{2}\right)\times 100$$ (1)

$$\text{LC}(\%)=\left(m_{1}/\;m_{3}\right)\times 100$$ (2)

### 2.9 *In vitro* drug release and stability studies

The dialysis method was used to simulate the release of curcumin from PLGA@Lipid NPs *in vitro*. Briefly, 2 mL of Cur@PLGA@Lipid NPs were placed into dialysis bags (MWCO = 10 kDa) and incubated in 100 mL of pre-warmed PBS solution containing 20% anhydrous ethanol (at pH 5.5 and 7.4) with gentle shaking (100 rpm) at 37 °C. We acknowledge that the use of 20% ethanol is a methodological limitation. Ethanol may disrupt the integrity of both the PLGA polymer matrix and the lipid bilayer, leading to artificially accelerated release compared to physiological conditions. This approach was adapted from a study to maintain sink conditions given curcumin’s poor aqueous solubility (Tabanelli et al., 2021). Accordingly, the release results are interpreted as comparative profiles between formulations, rather than absolute predictors of *in vivo* behavior. At specific time points, 2 mL of the released medium was collected and replaced with the same volume of fresh medium. The cumulative amounts of Cur in the 2 mL samples were determined by UV-vis spectrophotometry, and the cumulative release was calculated. The test was repeated three times, and all data were expressed as the mean ± standard deviation. The prepared NPs were stored at 4 °C for 1 month. The particle size and PDI were tested every 7 days to monitor sample stability.

### 2.10 Release kinetic modeling

To further evaluate the curcumin release mechanism, the cumulative release profiles of Cur@PLGA@Lipid NPs at pH 7.4 and pH 5.5 were fitted to zero-order, first-order, Higuchi, and Korsmeyer–Peppas kinetic models. The zero-order model was expressed as Qt = Q0 + k0t, where Qt is the cumulative amount of drug released at time t and k0 is the zero-order release constant. The first-order model was expressed as ln (100 − Qt) = ln100 − k1t, where k1 is the first-order release constant. The Higuchi model was expressed as Qt = kHt1/2, where kH is the Higuchi release constant. The Korsmeyer–Peppas model was expressed as Mt/M∞ = kKPtn, where Mt/M∞ is the fractional drug release at time t, kKP is the kinetic constant, and n is the release exponent. Model fitting was evaluated using the coefficient of determination (R^2^), and the release exponent n was used to infer the dominant release mechanism.

### 2.11 Cell culture

Human foreskin fibroblasts (HFF-1) and HeLa cells were purchased from the China Centre for Type Culture Collection (Shanghai, China). The cells were cultured in DMEM with 10% fetal bovine serum (FBS) and 1% penicillin/streptomycin at 37 °C and 5% CO_2_ in an incubator (Zi et al., 2025). HeLa cells were chosen as the cancer model due to their well-established relevance in cervical cancer research and their extensive use in NP-based therapeutic studies, enabling a controlled and focused proof-of-concept evaluation (Ahmed et al., 2025).

### 2.12 *In vitro* cytotoxicity assays

MTT assay was performed on HFF-1 and HeLa cells to evaluate any NP-induced cytotoxicity. Briefly, the cells were evenly seeded in a 96-well plate at a density of 5.0 × 10^3^ cells per well and cultured at 37 °C and 5% CO_2_ for 24 h (Santos et al., 2024). Subsequently, the cells were treated with free Cur, CuS, Cur@PLGA@Lipid NPs, and CuS/Cur@PLGA@Lipid with different concentrations of Cur (10–100 μg/mL) for 24 h. In addition, to evaluate the photothermal effect of CuS, 980 nm near-infrared (NIR) irradiation at 1.0 W/cm^2^ was applied for 5 min. After incubation, MTT solution (10 μL, 5 mg/mL) was added to each well and incubated for another 4 h at 37 °C. Then, the medium was removed and 150 μL DMSO was added as formazan solvent. The absorbance of the solution was measured at 570 nm the using a microplate reader (Varioskan LUX, Thermo Fisher, United States) to calculate the cell viability (Septisetyani et al., 2014). Each treatment was repeated six times in 96-well plates.

### 2.13 Live/dead cell staining

The live/dead cell staining was used for analysis of cytotoxicity. HeLa cells were seeded on cell culture plates at a density of 5.0 × 10^5^ cells/well and cultured for 24 h before being incubated with curcumin (Yao et al., 2024), Cur@PLGA@Lipid NPs, CuS/Cur@PLGA@Lipid NPs. In the NIR (+) group, all cells were irradiated with NIR (1.0 W/cm^2^, 5 min) at 980 nm wavelength after the cells were cultured for 24 h. Cells were washed twice with PBS, followed by incubation with DAPI (2 μg/mL) at 37 °C for 10 min and PI (5 μg/mL) for 20 min (Kølle et al., 2013). Then the cells’ morphology was observed under a fluorescence microscope (Nikon, Japan, Ti2-U, Tokyo, Japan). Blue stained cells represent all cells and red stained cells represent dead cells, respectively.

### 2.14 Cellular uptake studies

The experiment utilizes Cur’s natural green fluorescence to quantitatively assess the cellular drug uptake in HeLa cells via fluorescence microscopy. HeLa cells were plated in a 12-well plate and allowed to culture overnight. Curcumin, Cur@PLGA@Lipid NPs, and CuS/Cur@PLGA@Lipid NPs were introduced to the cells, which were then incubated for 1, 2, 4, 12, and 24 h, respectively. Then, the cells were washed, fixed with 4% paraformaldehyde for 10 min, and stained with DAPI (2 μg/mL) for 8 min, before being imaged with a fluorescence microscope (Lee et al., 2014).

Flow cytometry was used for quantitative determination of drug uptake. Briefly, the cells were trypsinized and washed three times with PBS before being analyzed by flow cytometry to analyze the internalization efficiencies of the cells. Data from a total of 10,000 events were acquired for each sample and the mean fluorescence intensity of Cur in the cells was analyzed using the Flowjo software.

### 2.15 Multicellular tumor spheroid (MCTS) assay

Agarose solution (1%) was added to a 96-well plate (100 μL/well) and allowed to solidify. HeLa cells were seeded in the 96-well plate at a density of 1 × 10^4^ cells per well for 7 days to form a 500 μm radius cell sphere (Komeri et al., 2022). The cell culture medium was subsequently replaced with fresh medium containing Cur, Cur@PLGA NPs, Cur@PLGA@Lipid NPs, CuS/Cur@PLGA@Lipid NPs, while PBS was used as the control. The (MCTSs) were imaged over a period of 3 days during treatment.

### 2.16 Statistical analysis

All values in this study are presented as means ± SD (standard deviation). Group differences were analysed using one-way analysis of variance (ANOVA) with GraphPad Prism 9.0 software. The number of repeated measurements for each experiment is indicated in the corresponding experimental sections. Statistical significance was defined as ****P < 0.0001; ***P < 0.001; **P < 0.01; and *P < 0.05.

## 3 Results

### 3.1 Synthesis and characterization of PLGA NPs

A microfluidic swirl mixer has been used to formulate the uniform NPs as drug delivery systems (DDSs). The device has shown excellent performance in nano formulations of lipids, polymer, protein and MOF NPs (Xu et al., 2022; Tomeh et al., 2022a; To et al., 2020; Tomeh et al., 2022b; Jin et al., 2023; Qiu et al., 2023; Mu et al., 2024; Zhang et al., 2025a; Pan et al., 2025; Lv et al., 2021). Figure 1 systematically illustrated the general workflow of the preparation processes and evaluates the influence of key microfluidic parameters—flow rate ratio (FRR, O/W), total flow rate (TFR), PLGA concentration, and Cur concentration—on the physicochemical properties of blank and Cur-loaded PLGA NPs.

The Cus/Cur@PLGA NPs were initially prepared by mixing the raw materials Curcumin and PLGA in solvent with CuS water solution. The HSPC/cholesterol solution in ethanol was subsequently mixed with the CuS/Cur@PLGA NP suspension to form the lipid coating on the CuS/Cur@PLGA NPs, yielding CuS/Cur@PLGA@Lipid NPs. The FRR (O/W) shown in Figure 1b have noticeable impacts on NP size and PDI. At a fixed TFR of 15 mL/min, increasing the FRR from 1:1 to 1:9 resulted in a decrease and then increase in particle size, with a minimal size observed at an FRR of 1:3 (Guo et al., 2023). The PDI exhibited a slight increase with higher FRRs, indicating reduced uniformity in particle size distribution at elevated ratios. The ζ potential of blank PLGA NPs in Figure 1c remained consistently negative across varying FRRs, showing minimal fluctuation and confirming stable surface charge.

Increasing the TFR from 5 to 60 mL/min progressively reduced the particle size (Figure 1d), with a small impact on PDI. This trend suggests reduced mixing time resulting in smaller size. Corresponding ζ potential values (Figure 1e) showed minimal variation across the tested TFR range, reinforcing the primary role of TFR in size control rather than surface charge modulation.

Increasing PLGA concentrations (2.5–20 mg/mL) progressively increased particle size from ∼50 to ∼130 nm, while the PDI slightly increased but still below 0.2, indicating good particle uniformity despite increased material content (Figure 1f). It was found that Cur concentration (Figure 1g) had a minor influence on particle size (0.2–0.5 mg/mL). However, as Cur concentration approached 0.8 mg/mL, particle size slightly increased, and the PDI exhibited a modest rise.

Collectively, these results highlight the intricate balance between formulation parameters and NP properties. Optimal conditions (FRR of 1:3, TFR of 15 mL/min, and moderate PLGA/Cur concentrations) enable the production of small, uniform, and stable PLGA NPs, reinforcing the utility of microfluidics for precision NP synthesis.

The size-controlling effect of the microfluidic swirl mixer can be explained by rapid mixing of solvent–antisolvent inside the mixing chamber. When the organic phase containing PLGA/curcumin meets the aqueous phase, the swirl geometry promotes transverse flow, rotational advection, and repeated stretching and folding of the fluid interface. This reduces the diffusion length between acetonitrile and water and accelerates local supersaturation of PLGA, favoring rapid and relatively homogeneous nucleation before extensive particle growth or aggregation occurs. From a nanoprecipitation perspective, increasing TFR increases convective mixing intensity and shortens the time available for polymer-rich domains to grow, which is consistent with the observed decrease in Cur@PLGA particle size from approximately 130 nm at 5 mL/min to approximately 60 nm at 60 mL/min. Similarly, the FRR controls the rate of solvent dilution and the final solvent composition; an FRR of 1:3 provided a balance between rapid supersaturation, stable nucleation, and limited aggregation, producing the smallest particle size with low PDI. Although direct computational fluid dynamics simulation or experimental mixing-time measurement was not performed in this study, the systematic reduction in particle size and maintenance of low PDI with optimized FRR/TFR provide experimental evidence that hydrodynamic mixing conditions strongly influence nanoprecipitation kinetics, which has been demonstrated in our previous report (Tomeh et al., 2022a).

### 3.2 Synthesis and characterization of lipid NPs

TFR has a significant effect on particle size (Figure 2a). Increasing the TFR from 5 to 60 mL/min progressively reduced the particle size, with minimal impact on PDI. This trend suggests reducing the mixing time improved mixing efficiency. Corresponding ζ potential values (Figure 2b) showed minimal variation across the tested TFR range, reinforcing the primary role of TFR in size control rather than surface charge modulation. The FRR between the aqueous (W) and organic (O) phases significantly impacts NP size and PDI (Figure 2c). At a fixed TFR of 40 mL/min, increasing the FRR from 1:1 to 9:1 increased particle size, with a minimal size observed at an FRR of 3:1 and 4:1. The stable PDI exhibited indicates robust uniformity across the FRRs. The ζ potential of the blank lipid NPs remained consistently negative across the tested FRRs (1:1 to 1:5) (Figure 2d), showing minimal fluctuation and confirming stable surface charge characteristics.

**FIGURE 2:**
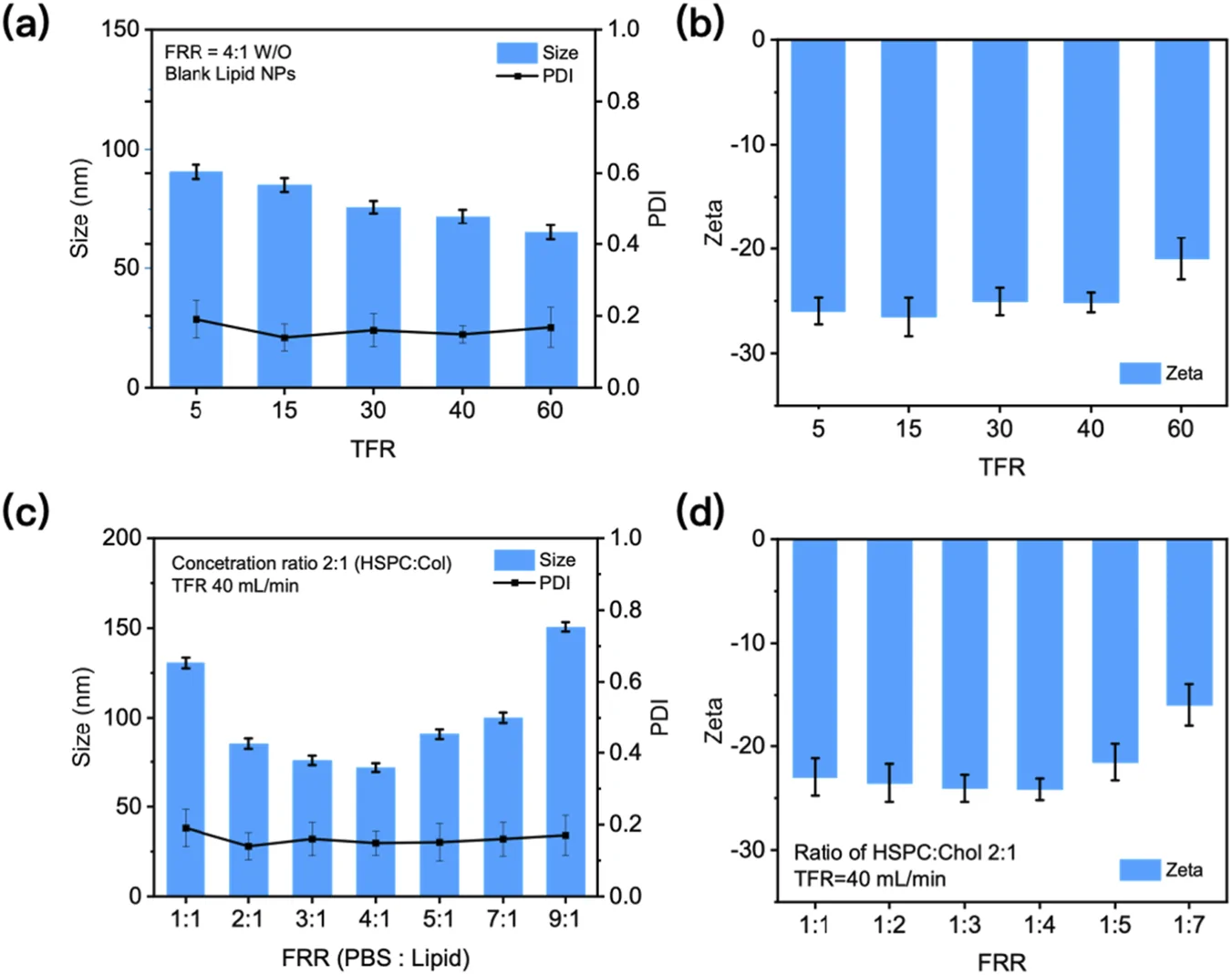
Optimization of lipid nanoparticles. Effects of total flow rate on particle size/PDI **(a)** and ζ potential. **(b)** Effects of flow rate ratio on particle size/PDI **(c)** and ζ potential. **(d)** Data are presented as mean ± SD (n = 3).

### 3.3 Synthesis and characterization of Cur@PLGA NPs

Figure 3a illustrates the effect of different FRR on the particle size and PDI of Cur@PLGA NPs. With the increasing FRR, the particle size decreased slightly before stabilizing at higher ratios. The reduction in particle size is attributed to improved mixing efficiency within the microfluidic system, which accelerates nanoprecipitation and enhances particle size control. The PDI values increased at high FRRs, indicating reduced uniformity at high FRRs. Among the tested conditions, an FRR of 1:3 produced the smallest particle size with lower PDI values, making it the optimal ratio for achieving well-defined NPs. As shown in Figure 3b, increasing the FRR has a clear effect on the ζ -potential of Cur@PLGA NPs. The ζ-potential values remained negative (below –20 mV) across all tested FRRs, indicating physical/colloidal storage stability. The ζ potential value at FRR 1:3 was sufficiently negative (−30 mV) to ensure particle repulsion and prevent aggregation, further supporting its selection as the optimal FRR.

**FIGURE 3:**
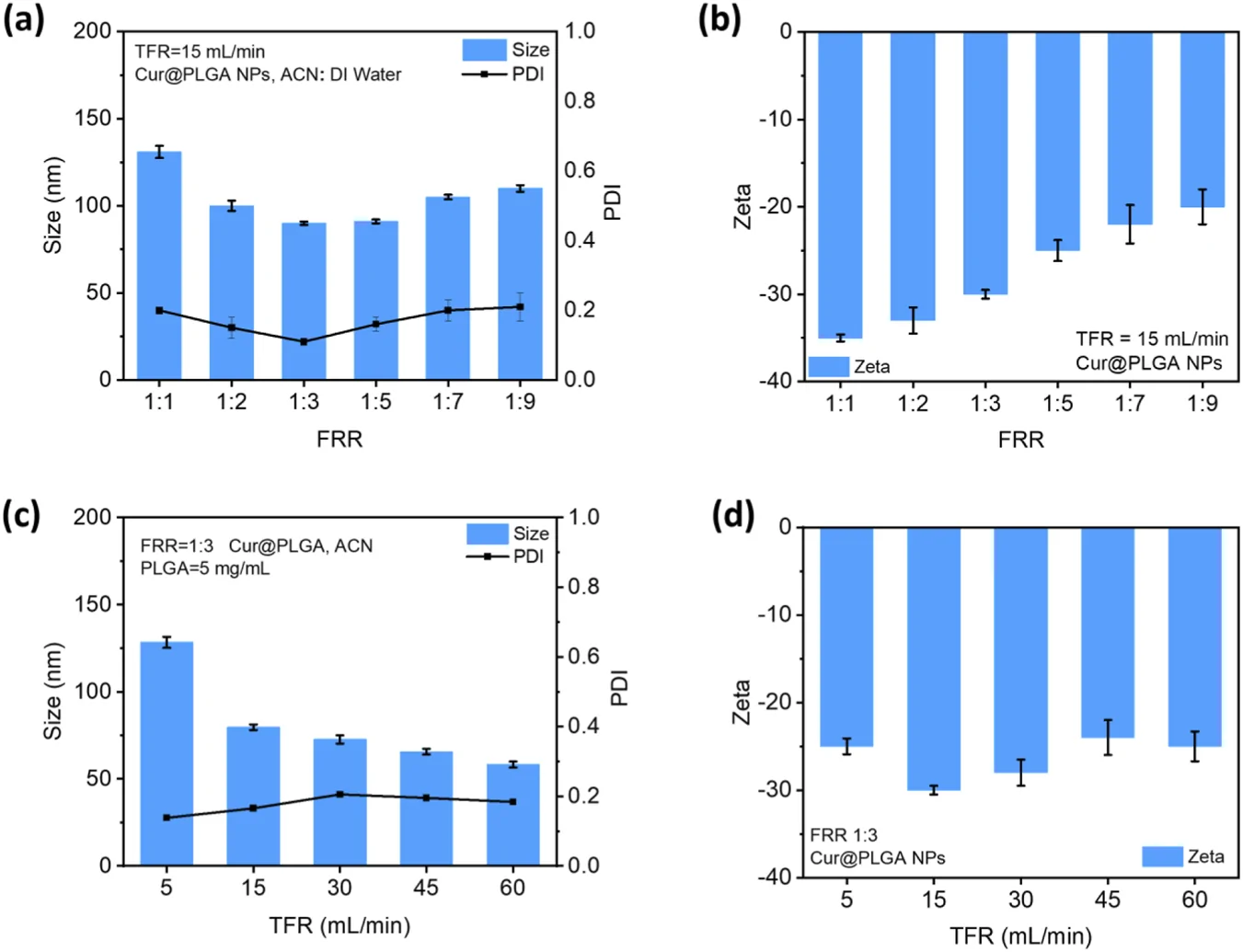
Optimization of Cur@PLGA nanoparticles. Effects of flow rate ratio on particle size/PDI **(a)** and ζ potential. **(b)** Effects of total flow rate on particle size/PDI **(c)** and ζ potential. **(d)** Data are presented as mean ± SD (n = 3).

Figure 3c presents the effect of different TFR on the particle size and PDI of Cur@PLGA NPs. Increasing the TFR resulted in a significant decrease in particle size until stabilization at higher flow rates. This reduction is attributed to enhanced mixing, which promotes faster nanoprecipitation and reduces particle growth and aggregation. A TFR of 15 mL/min produced a small particle size with a low PDI value, confirming its suitability for producing uniform NPs with favorable physical/colloidal storage stability. Figure 3d demonstrates the insignificant effect of TFR on the ζ potential of Cur@PLGA NPs. The stable ζ potential at TFR 15 mL/min (−30 mV) indicates that this condition effectively maintains the surface charge, contributing to improved colloidal stability. These results confirm that the combination of FRR = 1:3 and TFR = 15 mL/min provides the most favorable balance of small particle size, uniformity, and stability, supporting their selection as optimal conditions for synthesizing Cur@PLGA NPs via microfluidics. This optimization underscores the strength of microfluidic platforms in precisely tuning NP properties for enhanced drug delivery applications.

### 3.4 Structural evolution and size characterization of functionalized lipid nanoparticles

HSPC:cholesterol ratio at 2:1 was selected for the lipids formulation and PLGA NP coating as this composition offered the best balance of particle size and PDI (Korani et al., 2020). Figure 4 shows the size distribution of the NPs. The blank lipids NPs (Figure 4a) appear transparent with a narrow size distribution centered around 85 nm, indicating uniformity and successful lipid formation without additional payloads. After loading curcumin into PLGA, the resulting Cur@PLGA NPs (Figure 4b) acquire a distinct yellow color, reflecting the presence of curcumin, with an average diameter of 79 nm, slightly smaller than the blank lipids NPs, suggesting a compact NP structure. Further functionalization with lipids results in Cur@PLGA@Lipid NPs (Figure 4c), which exhibit a light-yellow hue and a size increase to approximately 115 nm, confirming effective lipid coating and efficient encapsulation. Finally, the addition of CuS to create CuS/Cur@PLGA@Lipid NPs (Figure 4d) imparts a yellow-green color, indicating successful CuS incorporation, with a further size increase to about 120 nm.

**FIGURE 4:**
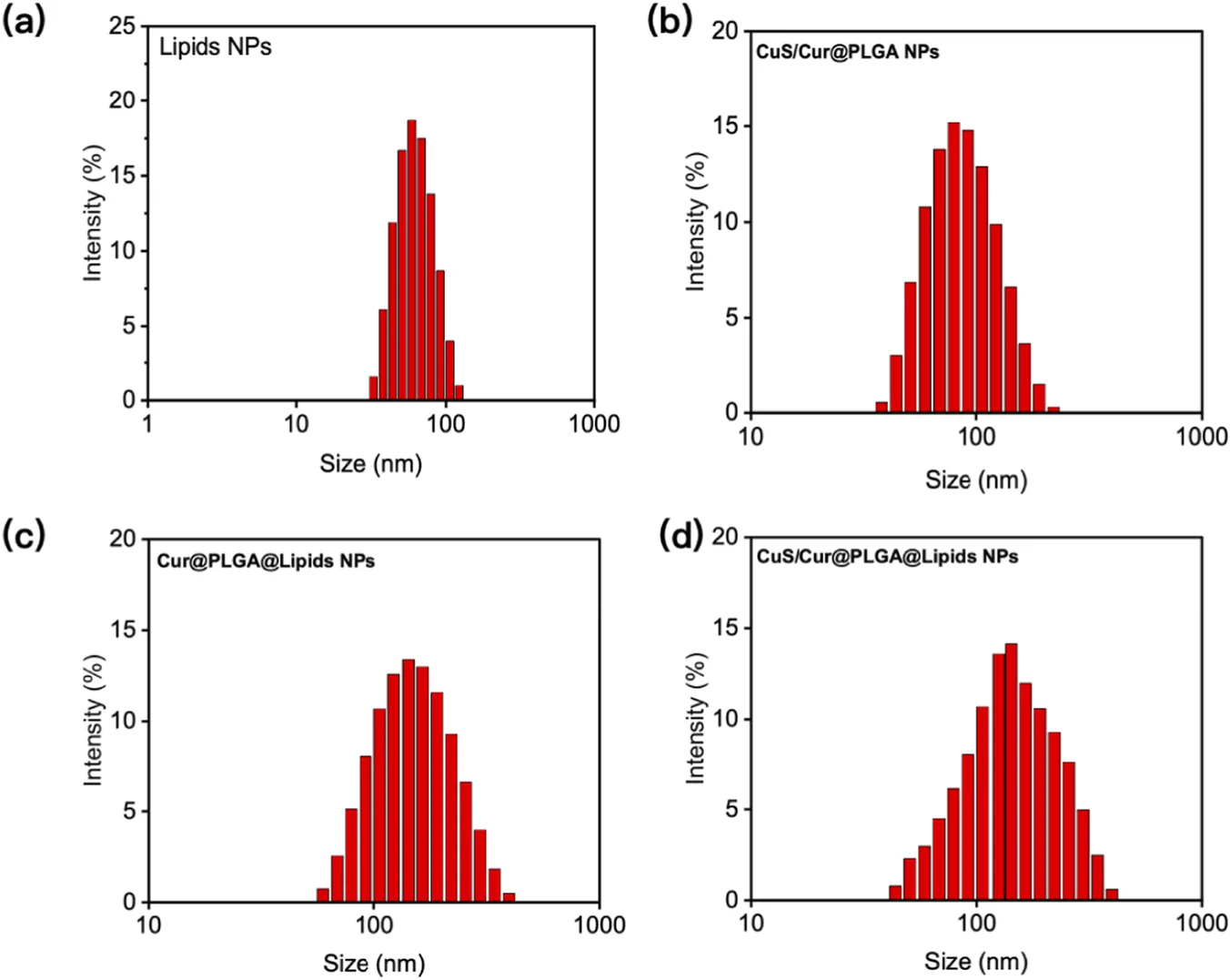
Particle size distribution of blank lipid NPs **(a)**, Cur@PLGA NPs **(b)**, Cur@PLGA@Lipid NPs **(c)**, CuS/Cur@PLGA@Lipid NPs **(d)**.

The increase in hydrodynamic diameter after lipid coating should not be interpreted as the thickness of a single conventional phospholipid bilayer. A typical lipid bilayer is only several nanometers thick, whereas the larger apparent size increase observed here likely reflects a mixed phospholipid/cholesterol interfacial layer, hydration/solvation effects, and possible lipid-rich domains adsorbed onto the PLGA surface. Therefore, the coating is more appropriately described as a mixed lipid/cholesterol layer associated with the PLGA nanoparticle surface rather than a simple uniform bilayer.

The adsorption of the phospholipid/cholesterol layer onto PLGA is expected to be driven mainly by non-covalent physicochemical interactions. Hydrophobic interactions and van der Waals forces between the PLGA matrix and the lipid acyl chains can promote lipid association with the polymer surface, while cholesterol may improve packing and reduce lipid-chain mobility within the interfacial layer. In addition, polar interactions between phospholipid headgroups and the PLGA surface, together with hydration of the outer phospholipid headgroups, may contribute to colloidal stabilization. The increasingly negative ζ potential after lipid coating and the maintenance of low PDI support the formation of a stable coated NP dispersion. However, the strength of these interfacial interactions was not directly quantified in this study by methods such as isothermal titration calorimetry, quartz crystal microbalance, or surface force measurements; therefore, the proposed stabilization mechanism should be considered an interpretation based on formulation behavior rather than a direct measurement of binding energy.

### 3.5 Characterization of PLGA-based NPs: stability, photothermal response, pH-sensitive drug release, and photothermal properties

Figure 5a shows the encapsulation efficiency (EE) and loading efficiency (LE) of CuS and Cur in the CuS/Cur@PLGA@Lipid NPs, with Cur exhibiting significantly higher EE and LE, potentially due to its better compatibility with the PLGA matrix. Figure 5b illustrates the particle size and PDI of various NP formulations. The particle size progressively increases from approximately 50 nm for PLGA NPs to around 130 nm for the fully loaded and coated NPs, indicating successful sequential loading and lipid coating. This value aligns with the dimensions observed in the AFM image in Supplementary Figure S1, in which the individual NPs appear to be 130 ± 5 nm in diameter, demonstrating consistency between DLS and AFM results. PDI values remain below 0.2, suggesting a uniform particle distribution. Figure 5c displays the ζ- potential of the same formulations. The surface charge becomes increasingly negative, from approximately −20 to −35 mV, as more components are incorporated and the lipid shell is added, indicating enhanced stability due to increased electrostatic repulsion. The transmission electron microscope (TEM) image showed the coated PLGA NPs and the CuS particles as the dots (Figure 5d). Since the size was larger than the bilayer-only pattern, the coating is presumed to comprise mixed phospholipid/cholesterol layers surrounding the PLGA NPs. Supplementary Figures S2a,b demonstrate the stability of CuS/Cur@PLGA@Lipid NPs stored in PBS and H_2_O at 4 °C and 37 °C over 1 month. Minimal changes in particle size and PDI indicate good physical stability in protein-free aqueous media under storage and temperature-controlled conditions.

**FIGURE 5:**
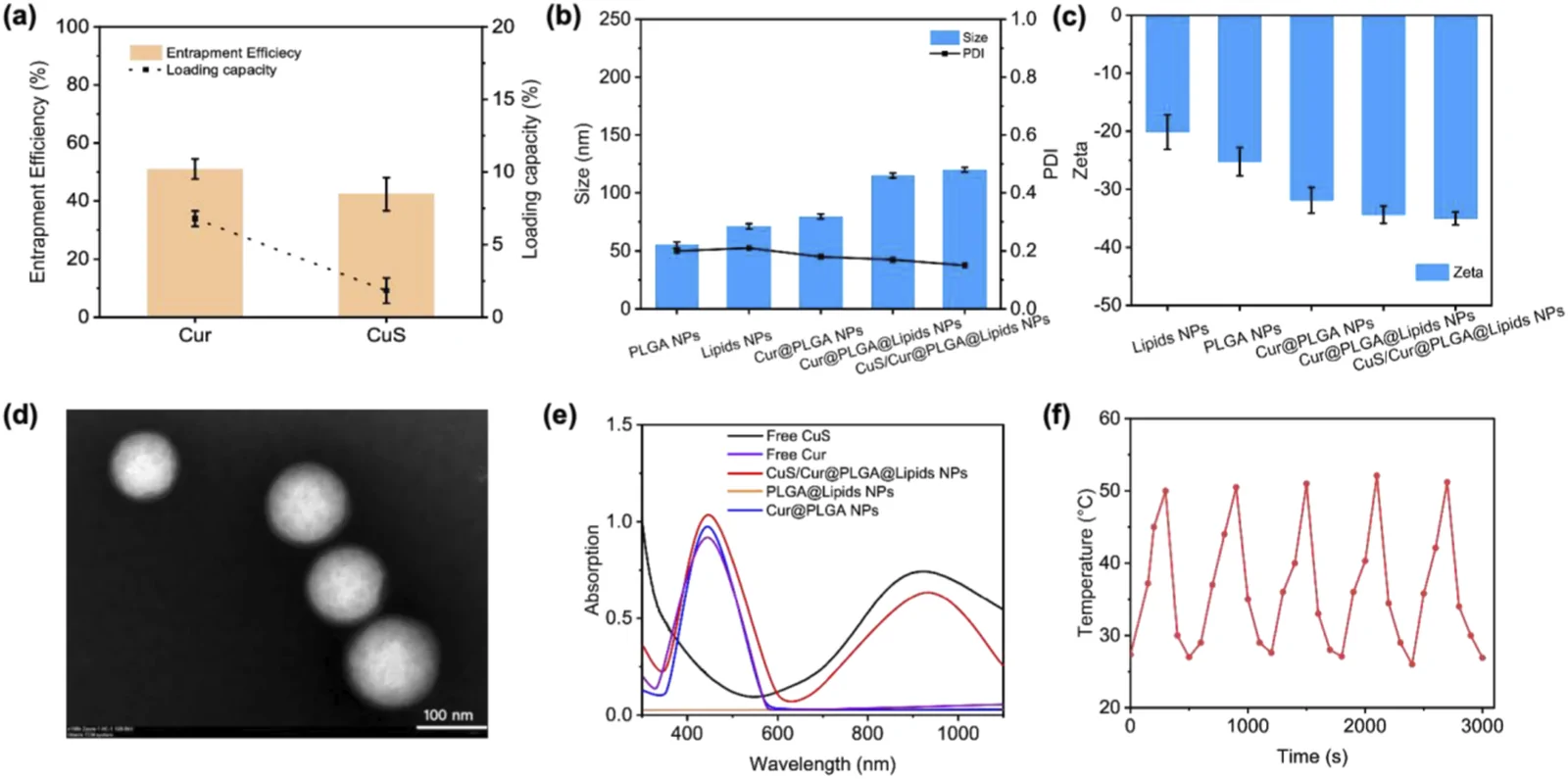
Characterization of CuS/Cur@PLGA@Lipid NPs. **(a)** Encapsulation efficiency and loading efficiency of curcumin and CuS. **(b)** Particle size and PDI of different formulations. **(c)** ζ potential of different formulations. **(d)** TEM image of CuS/Cur@PLGA@Lipids nanoparticles. **(e)** UV–Vis absorption spectra. **(f)** Photothermal stability during repeated laser on/off cycles. Data are presented as mean ± SD (n = 3).

The particle size remains essentially unchanged (≈80–95 nm) at 4 °C, with a slight increase at 37 °C, and PDI stays below 0.2 throughout, demonstrating that the NPs maintain their uniformity and resist aggregation in both pure water and PBS under storage temperatures. It should be noted that the present stability assessment was performed in protein-free media and does not directly predict nanoparticle behavior in biologically complex fluids. In serum-containing environments, adsorption of plasma proteins may alter nanoparticle size, surface charge, aggregation behavior, cellular uptake, biodistribution, and clearance through protein corona formation. Therefore, although the current data support physical colloidal stability during storage, additional studies in serum-containing media will give additional information in biological colloidal stability and protein corona effects before making conclusions about *in vivo* behavior.

Figure 5e shows the UV–Vis absorption spectra of the NPs. Distinct peaks at approximately 420 and 980 nm confirm the successful encapsulation of curcumin and incorporation of CuS, respectively, validating their chemical identity and potential for photothermal and therapeutic applications. The presence of CuS in the final formulation was supported by the characteristic near-infrared absorbance around 980 nm and by electron-dense features observed in TEM images. However, these techniques confirm CuS incorporation at the formulation level rather than its exact spatial distribution within the polymer–lipid architecture. We acknowledge that an EDS elemental mapping would give more detailed information of CuS location and distribution within the NPs. Based on the preparation sequence, in which CuS was introduced during PLGA nanoprecipitation before the lipid-coating step, CuS is presumed to be primarily associated with the PLGA NP phase. Supplementary Figure S3 depicts the pH-dependent cumulative release of curcumin from the NPs. A significant challenge in evaluating the release of hydrophobic drugs like curcumin is their poor aqueous solubility, which can lead to precipitation in the release medium and prevent accurate quantification (Carolina Alves et al., 2019). To overcome this, and to ensure appropriate ‘sink conditions’ as employed in previous studies, the release medium consisted of PBS containing 20% ethanol (Zhang et al., 2025b). Consistent with the study’s limitations, we emphasize that 20% ethanol is not representative of physiological fluids and may artificially enhance release by disrupting polymer and lipid structure (Nsairat et al., 2024). This approach was selected exclusively to ensure sink conditions for curcumin, and the data should be interpreted as relative comparisons between NP types. Future work will employ release media that better mimic *in vivo* environments. The results clearly demonstrate two key trends: First, curcumin release was significantly higher and faster at an acidic pH of 5.5 compared to physiological pH 7.4, which is attributed to the accelerated degradation of the PLGA matrix in acidic environments. Second, the lipid coating acted as an effective diffusion barrier, leading to a more sustained release profile compared to the uncoated PLGA NPs at both pH values. This pH-sensitive and sustained-release behavior may be relevant to drug delivery under acidic conditions; however, the present data should be interpreted as comparative *in vitro* release profiles rather than a direct prediction of physiological release in the tumor microenvironment (Bai et al., 2022). Release kinetic modeling was performed to further interpret the curcumin release mechanism from Cur@PLGA@Lipid NPs under physiological and acidic conditions. As shown in Supplementary Table S1, the release profile showed the best fit to the Korsmeyer–Peppas model at both pH 7.4 and pH 5.5, with R^2^ values of 0.992 and 1.000, respectively. The release exponent n was 0.115 at pH 7.4 and 0.281 at pH 5.5, indicating that curcumin release was predominantly governed by Fickian diffusion. The higher cumulative release observed under acidic conditions suggests that acid-facilitated PLGA matrix destabilization or degradation may further enhance curcumin release at pH 5.5, although diffusion remains the dominant release mechanism according to the kinetic fitting.

The FTIR spectra of CuS, PLGA NPs, and the CuS/Cur@PLGA@Lipid composite NPs were analyzed to confirm the encapsulation of Cur and CuS within the PLGA NPs (Supplementary Figure S4). To confirm that the encapsulation process did not induce chemical degradation or unwanted reactions between the components, FTIR analysis was performed. The spectrum for pure curcumin shows its characteristic peaks, including those for phenolic O-H stretching and aromatic C=C vibrations. The spectrum for the final CuS/Cur@PLGA@Lipid NPs is essentially a superposition of the characteristic peaks of its individual components (Curcumin, PLGA, and lipid), with the broad peaks from the polymer and lipids dominating the signal. Crucially, the absence of new peaks or significant shifts in the positions of curcumin’s characteristic peaks indicates that the drug remains chemically intact after encapsulation and that the components are combined through physical interactions rather than covalent bonding. This confirms the chemical compatibility of the loaded drugs within the NP matrix. The structural assignment of the final formulation as a lipid-associated PLGA NP is supported by the sequential increase in hydrodynamic size after lipid coating, changes in ζ potential, AFM/TEM morphology, UV–Vis absorbance of curcumin and CuS, and FTIR spectral compatibility. However, due to the unavailability of high-resolution TEM and EDS elemental mapping the exact CuS spatial distribution, absolute copper content, surface elemental composition were not revealed.

Supplementary Figure S5a illustrates the time-dependent temperature increase of CuS/Cur@PLGA@Lipid NP dispersions (0.1 mg/mL CuS) under 980 nm NIR laser irradiation at varying power densities (0.0–1.0 W/cm^2^ for 300 s). Higher power densities resulted in greater temperature elevation, with 1.0 W/cm^2^ achieving a temperature exceeding 50 °C, surpassing the hyperthermia threshold. Supplementary Figure S5b evaluates the photothermal performance of CuS/Cur@PLGA@Lipid NPs at different CuS concentrations (0.05–0.4 mg/mL) under a fixed irradiation power (1 W/cm^2^). While 0.05 mg/mL showed minimal efficacy, concentrations of 0.1 mg/mL and above induced significant temperature rise, confirming 0.1 mg/mL as the optimal concentration for balancing therapeutic impact and safety. Finally, Figure 5f examines the photothermal stability of the optimized formulation (0.1 mg/mL CuS, 1.0 W/cm^2^) over repeated on/off laser cycles. The consistent temperature fluctuations during 300 s of irradiation demonstrate robust stability, supporting its suitability for repeated clinical applications. This systematic analysis supports the power-dependent and concentration-dependent photothermal responsiveness of the CuS-containing NPs. Although calculation of photothermal conversion efficiency would provide a more direct quantitative comparison with established photothermal agents, this value was not calculated due to the reliable η determination was not available. Specifically, accurate calculation requires the full heating and cooling temperature–time profiles, the cooling time constant, the heat contribution from the solvent and container, the absorbance of the nanoparticle dispersion at 980 nm, and the effective incident laser power. Therefore, the current photothermal results should be interpreted as evidence of NIR-responsive heat generation, concentration dependence, power dependence, and irradiation-cycle stability rather than as a quantitative comparison of photothermal conversion efficiency with other photothermal platforms.

### 3.6 *In vitro* cytotoxicity

To evaluate the biocompatibility of the NPs, cell viability was assessed in HFF-1 fibroblast cells using the MTT assay. After 24 h of incubation with concentrations ranging from 0 to 100 μg/mL, CuS NPs, Cur@PLGA NPs, and Cur@PLGA@Lipid NPs showed acceptable cytocompatibility under non-irradiated conditions, with HFF-1 cell viability consistently exceeding 85% as shown in Figure 6a. However, this assay did not evaluate photothermal toxicity in HFF-1 cells under NIR irradiation.

**FIGURE 6:**
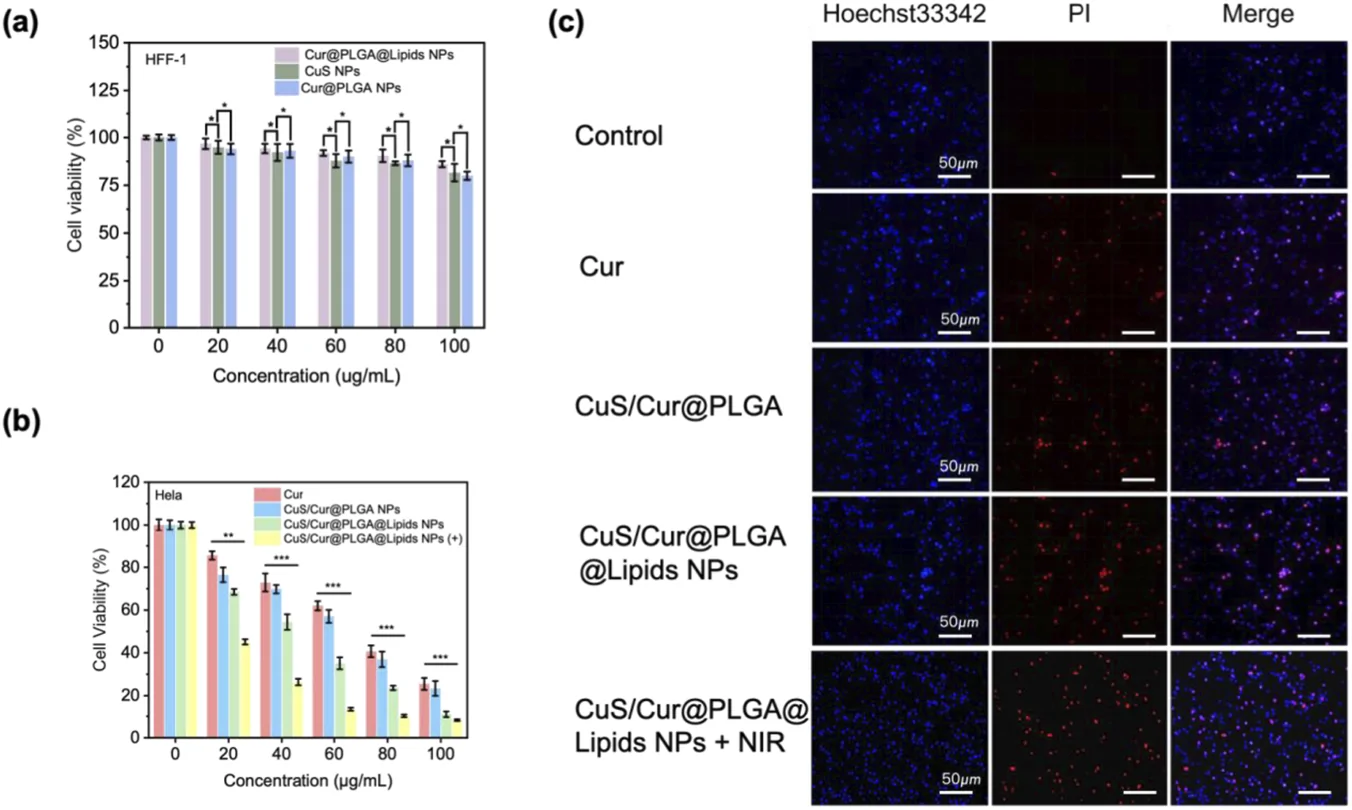
*In vitro* cytotoxicity and live/dead staining. **(a)** Viability of HFF-1 cells after treatment with blank NP formulations. **(b)** Viability of HeLa cells after treatment with curcumin-containing formulations. **(c)** Representative live/dead fluorescence images of HeLa cells after treatment. Blue indicates nuclei and red indicates dead cells. Data are presented as mean ± SD. Statistical significance is indicated as ****P < 0.0001; ***P < 0.001; *P < 0.01; and P < 0.05.

To assess the cytotoxicity of different NP formulations on cancer cells, HeLa cells were treated with various curcumin-containing formulations at concentrations between 20 and 100 μg/mL. As shown in Figure 6b, cell viability as analyzed using the MTT assay declined progressively with increasing concentrations of curcumin and NP formulations. Notably, CuS/Cur@PLGA@Lipid NPs with laser irradiation (+) exhibited the strongest cytotoxicity, achieving the lowest viability at higher concentrations. The enhanced cytotoxicity observed is likely related to a combination of controlled curcumin release from the PLGA matrix and improved toxicity from the photothermal effect of CuS after laser irradiation. Compared to the CuS/Cur@PLGA NPs, the lipid coating improved the intracellular accumulation therefore, improved the anticancer effect, although the specific uptake route responsible for this increased accumulation was not directly identified in the present study (Punfa et al., 2012). Given the limitations of the release assay performed in ethanol-containing media that may accelerate release, these results should be interpreted as comparative indicators of performance rather than absolute measures of sustained release–driven efficacy. Free curcumin, being a small amorphous molecule, is more susceptible to rapid efflux, resulting in lower intracellular accumulation and reduced efficacy. In contrast, curcumin encapsulated in CuS/Cur@PLGA@Lipid NPs is more efficiently internalized, which may be associated with enhanced nanoparticle internalization and intracellular retention, leading to enhanced improved therapeutic outcomes. To further explore the cytotoxic mechanisms, a live/dead assay was performed while blue fluorescence (Hoechst 33342) indicates the nuclei of all cells, and red fluorescence (propidium iodide, PI, red) indicates dead (Figure 6c). The CuS/Cur@PLGA@Lipid NPs with (+) and without laser irradiation exhibited strong red fluorescence, indicating increased cancer cell death, compared to Cur@PLGA NPs which consistent with the MTT results. Furthermore, the group treated with CuS/Cur@PLGA@Lipid NPs followed by 5 min of 980 nm NIR laser irradiation (1 W/cm^2^) showed the most intense red fluorescence and the highest level of cell death, highlighting the enhanced photothermal effect of CuS, which generates localized hyperthermia upon NIR irradiation, promoting thermal ablation of tumor cells. These findings demonstrate an combined chemo-photothermal cytotoxic effect of CuS/Cur@PLGA@Lipid NPs under NIR irradiation. However, the present assays provide phenotypic evidence of increased cell death rather than direct mechanistic confirmation of apoptosis, ROS generation, or mitochondrial membrane disruption. Because NIR-assisted photothermal toxicity was not tested in HFF-1 cells, the present study cannot determine whether the photothermal treatment selectively increases toxicity toward cancer cells while sparing normal fibroblasts. Therefore, the photothermal cytotoxicity results should be interpreted as evidence of enhanced antitumor activity in HeLa cells rather than as proof of tumor-selective photothermal safety.

Although the enhanced cytotoxicity observed following NIR irradiation is consistent with the combined effects of curcumin delivery and CuS-mediated photothermal heating, elucidating the specific downstream mechanisms underlying cell death was beyond the scope of the present study and was therefore not investigated. Additional assays, including Annexin V/PI staining, caspase activation, intracellular ROS detection using DCFH-DA, and mitochondrial membrane potential assessment using JC-1, would provide further insights into whether apoptosis, oxidative stress, or mitochondrial dysfunction contributes to the observed response. Therefore, the current findings should be interpreted as evidence of enhanced *in vitro* cytotoxicity following combined chemo-photothermal treatment rather than definitive mechanistic validation of the underlying cell-death pathways.

### 3.7 Cellular uptake experiments

The qualitative assessment of NP uptake by HeLa and HFF-1 cells was performed using inverted fluorescence microscopy, as shown in Figure 7. Cell nuclei were stained with DAPI (blue), while curcumin exhibited green fluorescence (Dutta et al., 2024). As seen in Figure 7a, the green fluorescence intensity gradually increased over time in both the Cur and CuS/Cur@PLGA@Lipid groups, indicating time-dependent internalization. Notably, the CuS/Cur@PLGA@Lipid consistently exhibited stronger fluorescence intensity than the Cur group at all observed time points, suggesting improved cellular uptake efficiency. Flow cytometry analysis (Figures 7b,c) quantitatively supported this observation, showing significantly higher mean fluorescence intensities in the CuS/Cur@PLGA@Lipid group, particularly at 6, 12, and 24 h.

**FIGURE 7:**
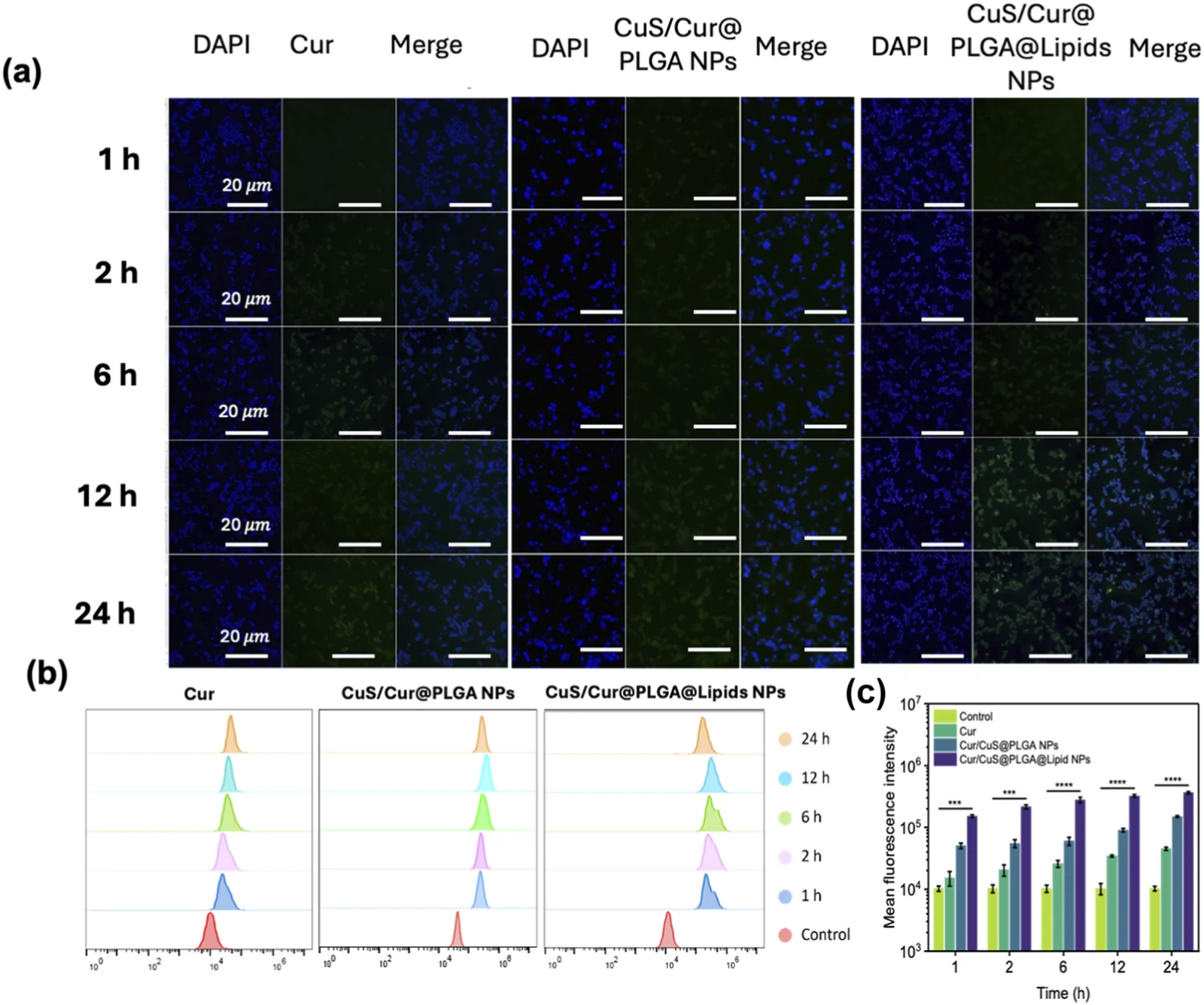
Cellular uptake of curcumin-containing formulations in HeLa cells. **(a)** Representative fluorescence microscopy images. **(b)** Representative flow cytometry histograms. **(c)** Quantitative flow cytometry analysis of intracellular curcumin fluorescence. Data are presented as mean ± SD. Statistical significance is indicated as ****P < 0.0001; ***P < 0.001; *P < 0.01; and P < 0.05.

The enhanced intracellular fluorescence observed for the lipid-coated formulation indicates improved nanoparticle-associated curcumin accumulation, but the precise internalization pathway was not determined. Lipid-coated polymeric nanoparticles may enter cells through multiple pathways, including clathrin-mediated endocytosis, caveolae/lipid raft-mediated uptake, macropinocytosis, or lipid membrane-associated interactions. Future studies using pharmacological inhibitors such as chlorpromazine for clathrin-mediated uptake, filipin or genistein for caveolae/lipid raft-mediated uptake, amiloride for macropinocytosis, and low-temperature uptake controls, together with endosomal/lysosomal co-localization imaging, would help define the dominant internalization route.

To verify that the fluorescence signal in Figure 7 originated specifically from curcumin, rather than interference by CuS, flow cytometry was configured to selectively detect curcumin fluorescence (excitation ∼425 nm, emission ∼530 nm), thereby excluding potential CuS signal contributions (CuS exhibits emission in the orange–cyan spectral range 585–605 nm) (Mogharbel et al., 2018). Importantly, a control group of CuS/PLGA@Lipid NPs without curcumin was analyzed and exhibited negligible fluorescence, confirming that the signals observed in Figure 7 indeed reflect curcumin uptake. Collectively, these results validate the specificity of the uptake data and reinforce the conclusion that the enhanced fluorescence observed in CuS/Cur@PLGA@Lipid NPs arises from improved curcumin internalization.

### 3.8 Spheroid assay analysis

Following the 2D cytotoxicity experiments, the anticancer potential of the prepared NPs was further evaluated using a 3D multicellular spheroid model composed of HeLa cells. The 3D tumor spheroid assay is an advanced *in vitro* method that better mimics the *in vivo* tumor microenvironment compared to traditional 2D monolayer cultures. This system replicates key tumor characteristics such as cell-cell interactions, oxygen, and nutrient gradients, providing a more realistic model for studying tumor growth, invasion, and drug response. As shown in Figure 8, the untreated and CuS groups maintained intact and compact spheroid structures throughout the experiment, indicating that CuS alone exhibited minimal cytotoxicity. Cur and Cur@PLGA NPs groups however, demonstrated a noticeable toxic effect. In contrast, the Cur@PLGA@Lipid group displayed serious disruption of the spheroid structure, demonstrating enhanced anticancer activity. Notably, the CuS/Cur@PLGA@Lipid NPs with laser group exhibited the most substantial spheroid degradation and cell death, as evidenced by the significant disintegration of the tumor spheroids. This pronounced effect highlights the superior anticancer efficacy of CuS/Cur@PLGA@Lipid NPs, which aligns with the findings from the 2D cell monolayer assays.

**FIGURE 8:**
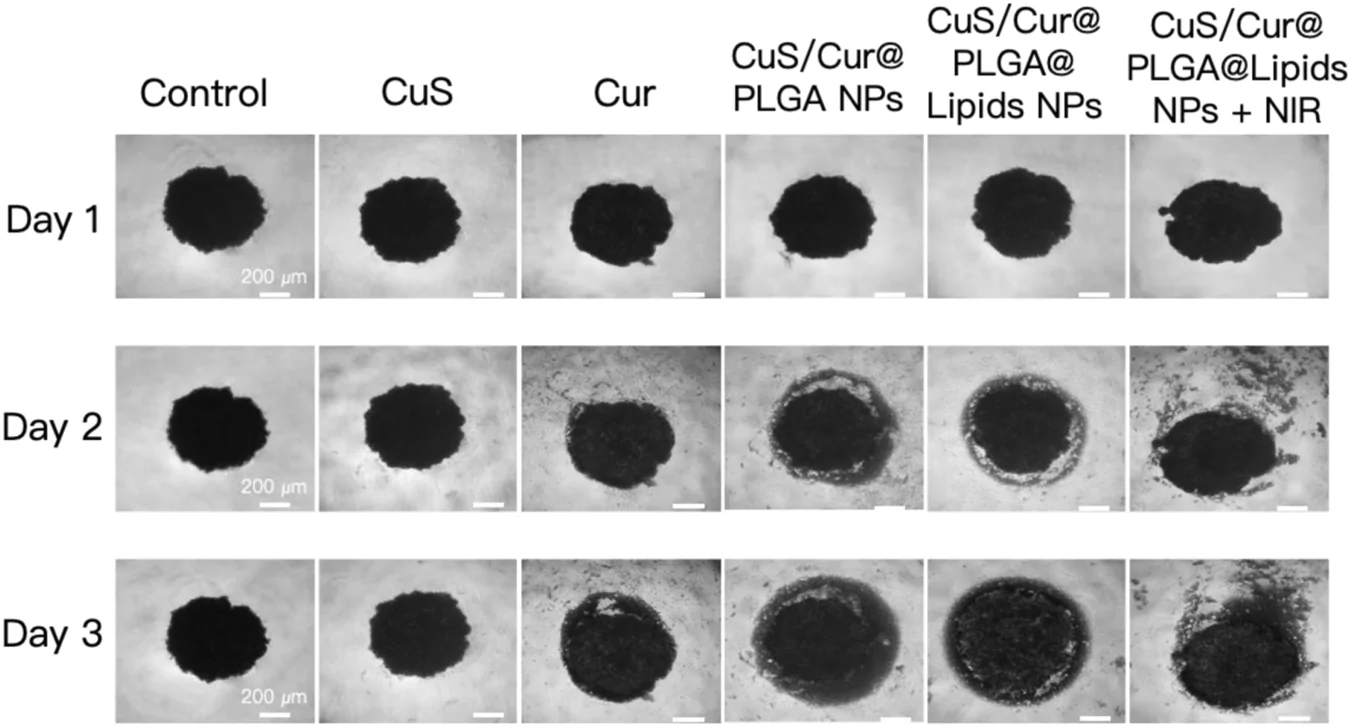
Antitumor activity of different formulations in HeLa multicellular tumor spheroids over 3 days of treatment. Images are representative of spheroid morphology and structural disruption.

The enhanced performance of CuS/Cur@PLGA@Lipid NPs (+laser) was associated with curcumin delivery, sustained release behavior, and CuS-mediated photothermal activity. However, quantitative nanoparticle penetration-depth analysis was not performed in the present study. Therefore, the spheroid results should be interpreted as evidence of enhanced overall antitumor activity in a 3D model rather than direct proof of improved intratumoral transport or deep spheroid penetration.

The 3D spheroid model provides a more physiologically relevant barrier than conventional 2D monolayer cultures by incorporating cell–cell interactions and diffusion gradients. In the present study, however, the spheroid experiments were designed to evaluate overall spheroid morphology and structural disruption rather than quantitatively assess nanoparticle penetration depth. Confocal z-stack imaging, radial fluorescence intensity profiling, and quantitative analysis of fluorescence distribution from the spheroid periphery to the core were not performed. Therefore, the enhanced spheroid disruption observed following treatment with CuS/Cur@PLGA@Lipid NPs under NIR irradiation cannot be specifically attributed to deeper intratumoral penetration. Future studies will quantitatively evaluate nanoparticle penetration depth and spatial distribution within spheroids to determine whether lipid coating enhances intratumoral transport and penetration.

## 4 Discussion

This study developed CuS/Cur@PLGA@Lipid NPs by integrating PLGA-based curcumin delivery, phospholipid/cholesterol surface modification, CuS-mediated photothermal activity, and swirl-type microfluidic fabrication. The optimized NPs exhibited a size of approximately 115–130 nm, PDI <0.2, and high curcumin encapsulation efficiency, demonstrating the feasibility of integrating these functions within a single NP formulation.

### 4.1 Microfluidic fabrication and nanoparticle characteristics

The swirl-type microfluidic mixer promotes rapid convective mixing and interfacial transport between the organic and aqueous phases, which can accelerate solvent exchange and establish relatively homogeneous supersaturation during nanoprecipitation. Such conditions favor rapid nucleation and can reduce uncontrolled particle growth. Consistent with this principle, variation of the flow rate ratio and total flow rate enabled systematic control of NP size and PDI, with the optimized formulation producing relatively uniform nanoparticles.

Our previous studies established the utility of the swirl-type microfluidic platform for high-throughput nanoparticle fabrication (Xu et al., 2022). The present study extends this platform to a multifunctional PLGA–lipid formulation incorporating curcumin and CuS. However, Reynolds number, mixing time, CFD analysis, and direct comparison with bulk nanoprecipitation or other microfluidic mixers were not performed. Therefore, the present results demonstrate the tunable formulation capability and high-throughput potential of the platform rather than definitive superiority over alternative fabrication methods. Similarly, although a throughput of up to 320 mL/min has been demonstrated for the platform previously (Xu et al., 2022), prolonged continuous production and batch-to-batch reproducibility of the present formulation at this throughput were not evaluated.

The increase in hydrodynamic size and change in ζ-potential following lipid incorporation, together with the fabrication sequence and morphological characterization, support the formation of a lipid-associated surface layer. Hydrophobic interactions and interfacial rearrangement of amphiphilic lipids during nanoparticle formation may contribute to association of HSPC/cholesterol with the PLGA surface. However, the available data cannot directly establish a uniform lipid bilayer, its thickness, or adsorption strength. Thus, the formulation is more appropriately described as lipid-coated PLGA NPs. High-resolution cryo-TEM or complementary surface-sensitive characterization would be required for direct structural confirmation.

### 4.2 Curcumin release and cellular uptake

The optimized formulation achieved high curcumin encapsulation and exhibited sustained, pH-dependent *in vitro* release. Approximately 75%–80% of curcumin was released over 96 h at pH 7.4 from lipid-coated PLGA NPs, compared with approximately 60%–65% from PLGA-only NPs, while release was accelerated under acidic conditions. The altered release profile may arise from changes in interfacial hydration, matrix accessibility, and transport pathways associated with the lipid layer.

However, the present data do not distinguish quantitatively between drug diffusion, polymer degradation, and changes in matrix structure. In addition, curcumin was quantified by UV–Vis spectroscopy and the release conditions do not fully reproduce the physiological environment. Therefore, these results should be interpreted as evidence of sustained and pH-dependent *in vitro* release, rather than definitive proof of controlled *in vivo* release. HPLC analysis and release-kinetic modelling would provide additional mechanistic information in future studies. These findings are consistent with recent developments in stimuli-responsive liposomal and polymeric nanocarrier systems, which further support the potential of engineered nanocarriers for controlled therapeutic delivery (Basak et al., 2024; Basak and Das, 2026; Basak and Das, 2025a; Basak and Das, 2025b).

Fluorescence imaging and flow cytometry demonstrated enhanced intracellular accumulation for the lipid-coated formulation. This may result from altered NP–cell interactions and increased cellular internalization associated with the lipid-associated surface. However, specific endocytic pathways were not investigated; therefore, the current data support enhanced uptake but do not establish a particular internalization mechanism. The increased intracellular accumulation likely contributes to the improved biological response, although the cytotoxicity should not be attributed solely to the sustained-release behavior.

### 4.3 Photothermal activity and chemo-photothermal response

CuS incorporation introduced NIR-responsive photothermal functionality into the NP system. The CuS-containing NPs produced rapid temperature elevation under 980 nm irradiation and retained their photothermal response during repeated irradiation, indicating favorable photothermal stability under the tested conditions.

NIR irradiation further enhanced the cytotoxicity of CuS/Cur@PLGA@Lipid NPs against HeLa cells, consistent with the combined effects of curcumin delivery and CuS-mediated photothermal heating. The present data demonstrate an enhanced chemo-photothermal response, but do not establish a specific molecular mechanism or quantitative synergistic interaction. Mechanistic assays involving apoptosis, ROS generation, caspase activation, and mitochondrial membrane potential would be helpful to define the downstream cell-death pathways.

Photothermal conversion efficiency was also not calculated because the complete dataset required for reliable determination was unavailable. Therefore, photothermal performance was evaluated based on temperature elevation and irradiation stability rather than a standardized conversion-efficiency value.

### 4.4 Three-dimensional spheroid response

The 3D spheroid experiments provided a more structurally complex *in vitro* model than conventional 2D monolayer cultures. CuS/Cur@PLGA@Lipid NPs combined with NIR irradiation produced the greatest spheroid disruption, consistent with the enhanced cytotoxicity observed in 2D cultures.

However, the spheroid experiments assessed overall morphology and structural disruption rather than quantitative NP penetration. Therefore, the enhanced response cannot be specifically attributed to deeper penetration of lipid-coated NPs. Confocal z-stack imaging and spatial fluorescence analysis would be helpful to determine whether lipid coating alters nanoparticle transport from the spheroid periphery toward the core.

### 4.5 Significance and limitations

The main contribution of this study is the integration of microfluidic fabrication, lipid-associated PLGA NPs, curcumin delivery, and CuS-mediated photothermal activity within a single formulation strategy. The resulting NPs demonstrated favorable physicochemical properties, high curcumin encapsulation, sustained and pH-dependent *in vitro* release, enhanced cellular uptake, and NIR-enhanced cytotoxicity in both 2D cells and 3D spheroids.

Nevertheless, several limitations should be considered. TEM-EDS/STEM-EDS mapping, XPS, ICP would be helpful confirm the precise spatial localization of CuS within the NP architecture, while the TGA analyses would provide information regarding the thermal behavior and relative composition of the polymer/lipid-containing formulations. Serum stability and protein corona formation were also not investigated. In addition, the study used a single cancer cell line and did not evaluate photothermal toxicity of the complete formulation in normal HFF-1 fibroblasts. Quantitative mixing characterization, prolonged high-throughput manufacturing, batch-to-batch reproducibility, specific endocytic mechanisms, and quantitative spheroid penetration were also beyond the scope of the present work.

Overall, the findings provide an *in vitro* proof-of-concept for a multifunctional PLGA–lipid/CuS nanocarrier fabricated using a high-throughput microfluidic approach. The platform demonstrates potential for further development of combination chemo-photothermal nanomedicine, while additional physicochemical, mechanistic, manufacturing, and *in vivo* studies will be required to establish its broader translational potential.

## 5 Conclusion

In this study, CuS/Cur@PLGA@Lipid NPs were fabricated using a microfluidic swirl mixing approach for combined chemo-photothermal therapy. The optimized NPs exhibited a size of approximately 115–130 nm and a PDI below 0.2, together with high curcumin encapsulation efficiency and pH-dependent release behavior. Approximately 75%–80% of the encapsulated curcumin was released over 96 h at pH 7.4, with accelerated release observed under acidic conditions (pH 5.5). The NPs demonstrated enhanced cytotoxicity against HeLa cells, which was further increased by CuS-mediated photothermal heating under NIR irradiation. The formulation also exhibited favorable physical/colloidal storage stability and stable photothermal performance under repeated irradiation cycles.

The microfluidic platform provides a promising approach for the controlled fabrication of multifunctional NPs and offers potential advantages for high-throughput production. The phospholipid/cholesterol-associated surface layer may provide an alternative strategy to conventional PEGylation for modifying the interfacial properties of PLGA NPs, although direct comparative studies are required to establish this potential advantage. Overall, the present findings demonstrate the feasibility of integrating microfluidic fabrication, lipid-associated surface modification, curcumin delivery, and CuS-mediated photothermal therapy within a single multifunctional nanoplatform. While the enhanced *in vitro* chemo-photothermal cytotoxicity and favorable physicochemical properties support the potential of this platform for potential combination cancer therapy, further studies using additional cancer models, normal cells, and *in vivo* systems are required to validate its therapeutic efficacy, safety, and translational potential.

## Data Availability

The raw data supporting the conclusions of this article will be made available by the authors, without undue reservation.
