# Supplementary material for: Microfluidic fabrication of lipid-coated PLGA nanoparticles co-delivering CuS and curcumin for combined chemo-photothermal therapy
Source: Front Bioeng Biotechnol. 2026 Oct 28;14:1805754. doi: 10.3389/fbioe.2026.1805754 (PMC13751387; doi:10.3389/fbioe.2026.1805754)
Supplement: Supplementary file 1 [file Supplementaryfile1.docx]

Supplementary Materials

**Microfluidic Fabrication of lipids coating PLGA Nanoparticles Co-delivering CuS and Curcumin for Combined Chemo-Photothermal Therapy**

Alhasan Mohammed ^1^, Yu Xia ^1^, Hemin Roghzai ^2^, Bo Zhang ^1^, and Xiubo Zhao ^1^ *

^1^	School of Pharmacy, Changzhou University, Changzhou 213164, China

^2^	College of Science, University of Sulaimani, Sulaymaniyah 46001, Iraq

*****	Correspondence: xiubo.zhao@cczu.edu.cn

Supplementary Figures

Figure S1. AFM images of Cur@PLGA@Lipids NPs.

Figure S2. Stability of Cur@PLGA@Lipids NPs stored at 4 °C and 37 °C over one month in PBS media (a) and water (b), evaluated by monitoring particle size and PDI. Data are presented as mean ± SD from repeated measurements.

Figure S3. pH-dependent release profiles of curcumin, illustrating potential for sustained drug delivery. Data are presented as mean ± SD from repeated measurements.

Figure S4. FTIR spectra of CuS, Cur, Cur@PLGA NPs, and CuS/Cur@PLGA@Lipids NPs. The main characteristic FTIR bands are assigned in the caption to improve readability. Curcumin-related bands include broad O–H stretching, C=O/C=C stretching, aromatic C=C vibration, and C–O/C–O–C stretching. PLGA-related bands include ester C=O and C–O–C vibrations, while the lipid-coated formulation shows additional lipid-associated C–H, P=O, and P–O–C/C–O bands, supporting successful formation of the composite nanoparticle system.

Figure S5. Photothermal heating profiles of CuS/Cur@PLGA@Lipids NPs under 980 nm laser irradiation at different power densities (a) and temperature elevation at varying CuS concentrations (b). Data are presented as mean ± SD from repeated measurements.

Supplementary Table

Table S1. Kinetic modeling of curcumin release from Cur@PLGA@Lipids NPs

| **Model** | **Parameter** | **pH 7.4** | **pH 5.5** |
| --- | --- | --- | --- |
| Zero-order | R² | 0.659 | 0.589 |
| First-order | R² | 0.736 | 0.718 |
| Higuchi | R² | 0.873 | 0.839 |
| Korsmeyer–Peppas | kKP | 0.418 | 0.279 |
| Korsmeyer–Peppas | n | 0.115 | 0.281 |
| Korsmeyer–Peppas | R² | 0.992 | 1.000 |

Abbreviations: kKP, Korsmeyer–Peppas release rate constant; n, release exponent; R², coefficient of determination.

Table S2. Quantitative performance summary and positioning of the present platform

| **Feature** | **Present platform** | **Interpretation** |
| --- | --- | --- |
| Fabrication strategy | Swirl-type microfluidic fabrication with FRR/TFR optimization | Controlled preparation of PLGA–lipid hybrid nanoparticles |
| Therapeutic components | Curcumin + CuS | Combined chemo-photothermal functionality |
| Particle size | Approximately 115–130 nm | Suitable nanoscale range for cellular delivery studies |
| PDI | <0.2 | Narrow particle-size distribution |
| Encapsulation | Curcumin EE >80% | Efficient loading of hydrophobic drug |
| Release behavior | 75–80% cumulative release over 96 h at pH 7.4; accelerated release at pH 5.5 | Sustained and pH-responsive release |
| Release kinetic model | Korsmeyer–Peppas best fit; R² = 0.992 at pH 7.4 and 1.000 at pH 5.5 | Predominantly diffusion-controlled release |
| Photothermal behavior | NIR-responsive, power- and concentration-dependent heating | CuS-mediated photothermal function |
| Biological performance | Enhanced uptake and cytotoxicity in HeLa 2D/3D models | Improved in vitro performance compared with control formulations |
